# Participation in physical activities for children with cerebral palsy: feasibility and effectiveness of physical activity on prescription

**DOI:** 10.1186/s40945-017-0041-9

**Published:** 2017-11-28

**Authors:** Katarina Lauruschkus, Inger Hallström, Lena Westbom, Åsa Tornberg, Eva Nordmark

**Affiliations:** 1 Department of Health Sciences, Faculty of Medicine, Lund University, Box 157, -221 00 Lund, SE Sweden; 2 Department of Clinical Sciences Lund, Division of Paediatrics, Lund University, Lund, Sweden

**Keywords:** Children, Cerebral palsy, Participation, Physical activity, Physical activity on prescription, Sedentary behaviour

## Abstract

**Background:**

Children with cerebral palsy (CP) are less physically active and more sedentary than other children which implies risk factors for their physical and mental health. Physical activity on prescription (PAP) is an effective intervention to promote a lifestyle change towards increased physical activity in adults in general. Knowledge is lacking about the use of PAP in children with CP. Therefore, the aim of this study was to evaluate the feasibility of PAP for children with CP and its effectiveness on participation in physical activity and sedentary behaviour.

**Methods:**

Eleven children with CP, aged 7-11 years, participated in PAP, consisting of a written agreement between each child, their parents and the physiotherapist and based on Motivational Interviewing (MI), Canadian Occupational Performance Measure (COPM) and Goal Attainment Scaling (GAS). Individual goals, gross motor function and physical activity were assessed at baseline, at 8 and/or 11 months using COPM, GAS, logbooks, Gross Motor Function Measure (GMFM-66), physical activity questionnaires, physical activity and heart rate monitors and time-use diaries. At 8 and 11 months the feasibility of the intervention and costs and time spent for the families and the physiotherapist were evaluated by questionnaires.

**Results:**

The intervention was feasible according to the feasibility questionnaire. Each child participated in 1-3 self-selected physical activities during 3-6 months with support from the physiotherapist, and clinically meaningful increases from baseline of COPM and GAS scores were recorded. Being physically active at moderate-vigorous levels varied between less than 30 and more than 240 minutes/day, and the median for the whole group was 84 minutes/day at baseline and 106 minutes/day at 8 months.

**Conclusions:**

The intervention PAP seems to be feasible and effective for children with CP, involving both every day and organised physical activities to promote an active lifestyle through increased participation, motivation, and engagement in physical activities. Further research of PAP is needed, preferably in a long term randomised controlled trial and including health economic analysis to show costs and benefits.

**Trial Registration:**

ISRCTN76366356, retrospectively registered.

## Background

It is a challenge for children to meet the global recommendations of 60 minutes of moderate to vigorous physical activity per day [1], especially for those with cerebral palsy (CP), which is the most common physical disability in childhood [2]. Approximately 2-2.5/1000 children have CP with affected muscle tone, movement and motor skills, often accompanied by intellectual, communication, and behavioural impairment, as well as epilepsy and pain [2, 3]. Although the gross motor function between children with CP is extremely variable, the energy expenditure and muscle activity in children with CP elevate during standing, with or without support, compared to sitting, across all gross motor function levels [4]. Physical activity is defined as ‘any bodily movement produced by skeletal muscles that results in energy expenditure’ [5], while sedentary behaviour includes any waking behaviour characterised by little physical movement and low energy expenditure while in a sitting or reclining posture [6, 7]. Children with CP have more sedentary time and participate less in habitual physical activities than their peers without disabilities, which implies risks for health outcomes, physical function and human metabolism [8–11]. Additional to being and staying physically active, replacing sedentary time with light physical activity might be a beneficial way to reach health benefits for children with CP, especially for those with severe motor impairments when physical activity with moderate to vigorous intensity often is a huge challenge [12–15]. There is an association of reduced physical activity behaviour and elevated blood pressure values in children with CP [16]. An active lifestyle with spontaneous and organised physical leisure activities is recommended [17–19].

Children with CP want to be physically active, have fun and enjoy the sensation of speed [20]. Family preferences and attitudes towards exercise were described as cultural factors which were facilitating or hindering for participation in physical activities for children with CP. Other factors were social and financial support, as well as transportation and access to information about activities [20–22]. Parents and children with CP perceived attitudes at school and in the community, lack of personal skills, unfamiliar instructors when attending group activities and difficulties with accessing personal equipment as general barriers to participation [23, 24]. Each family’s interdependence and interaction between themselves and their environment should be considered when providing interventions for children with CP [25].

Childhood and adolescence are critical periods when self-image, attitudes and behaviours are developed that might be transferred into adulthood and interventions should focus on changeable behaviours and objectives [26]. Effectiveness studies aim to evaluate success in real-world conditions and in community-based settings [27]. Physical activity on prescription (PAP) is shown to be an effective intervention to promote a lifestyle change towards increased physical activity in adults [28]. It consists of a personalised prescription and can comprise of a written suggestion for structured facility-based activities as group activities or community-based activities such as walking, with or without a supportive structure, from a prescriber. All licensed health care professionals with adequate expertise may prescribe PAP [28]. Little is known about the effectiveness of PAP in children, especially in those with disabilities. For children with CP, exercise programmes, home-based physiotherapy and counselling, aiming to increase physical activity, did not yield long-term effects [29–31]. More knowledge is needed about interventions to promote a more active and health-enhancing lifestyle for children with CP in real-life settings, and about the ways in which physical activity can be positively viewed and encouraged among families with different cultural backgrounds and attitudes towards exercise. Therefore, the aim of this descriptive, exploratory study was to evaluate the feasibility of PAP for children with CP and its effectiveness on participation in physical activity and sedentary behaviour.

## Methods

### Participants

Eleven of 27 eligible children with CP were selected to participate in the study to represent different demographic and clinical groups. Six girls and five boys, aged 7-11 years at baseline, with various gross and fine motor, communicative and cognitive functions and from both rural areas and cities in the south of Sweden were included. The 18 parents of the children, aged 36-64 years, were also included; nine of them were born outside of Sweden, of which six came from non-European countries. Ten of the parents were employed as personal assistants for their child. The characteristics of the children and their parents are shown in Table 1.

**Table 1:** Characteristics of the children and their parents

Children with cerebral palsy (*N*=11)	n	Parents (*N*=18)	n
Gender		Gender	
Female	6	Female	10
Male	5	Male	8
Age (years)		Ethnic origin	
7	2	Sweden	9
8	1	Europe, other than Sweden	3
9	1	Outside Europe	6
10	4		
11	3	Language interpretation	2
GMFCS-E&R^a^ level		Marital status	
I	3	Married/cohabitant	16
II	2	Single parent	2
III	1		
IV	4	Children living in the household	
V	1	1	6
		2	8
MACS level^b^		3	4
I	4		
II	2	Level of education	
III	3	Secondary school	7
IV	1	University degree	11
V	1		
		Employment status	
CFCS level^c^		Working full time	12
I	5	Working part time	1
II	1	Studying	3
III	1	Unemployed	2
IV	1		
V	3	Working as personal assistant to the child	
		Main work	4
Cognitive level^d^		Part time	6
No mental retardation	6		
Mild mental retardation	1	Range of income before tax (in SEK^e^) per month/parent	6 400 – 85 000
Moderate to profound mental retardation	4		
Augmentative and alternative communication	4		
Language interpretation	1		

^a^Gross Motor Function Classification System Expanded and Revised; ^b^Manual Ability Classification System; ^c^Communication Function Classification System; ^d^WHO’s International Classification of Diseases (ICD): ICD-10 codes for mental retardation; ^e^Swedish crowns, 1 USD=8.88 SEK (annual average 2016)

### Study design

**Fig. 1:**
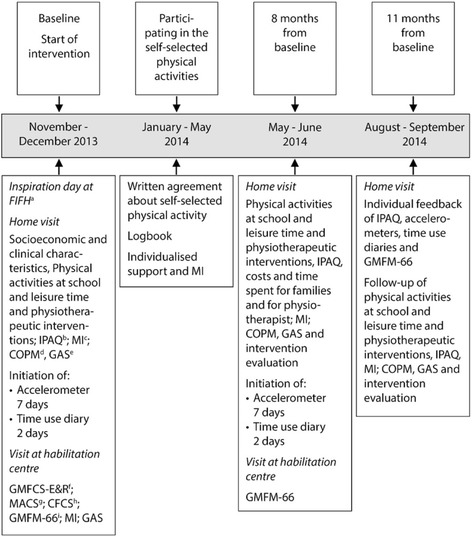
^a^FIFH (Association for Disability sports; www.fifh.com); ^b^IPAQ: International Physical Activity Questionnaire; ^c^MI: Motivational Interviewing; ^d^COPM: Canadian Occupational Performance Measure; ^e^GAS: Goal Attainment Scaling; ^f^GMFCS-E&R: Gross Motor Function Classification System; ^g^MACS: Manual Ability Classification System; ^h^CFCS: Communication Function Classification System; ^i^GMFM-66: Gross Motor Function Measure. Time line of the intervention including PAP for children with cerebral palsy

The feasibility and effectiveness of PAP for a lifestyle change were assessed within a real-life context and with an intervention period of three to six months. Questionnaires and outcome measures were conducted at baseline, and after 8 and 11 months (Fig. 1). Three physiotherapists (first and last author and a project assistant) with adequate training conducted the questionnaires and assessments and supported the families during the intervention.

### Physical activity on prescription

The intervention PAP comprised self-selected physical activities as a written agreement between each child, their parents and the physiotherapist, to enhance each child’s habitual physical activity and reduce sedentary behaviour. Motivational interviewing (MI) [32, 33] was used as a counselling method throughout the study period which enabled the physiotherapist to identify each child’s desire and to guide each child and their parents towards an active lifestyle. Individually designed logbooks enabled children to document their frequency of participation in their self-selected physical activities and to write comments.

#### Questionnaires and goal setting

Characteristics of each child and their parents were recorded in sociodemographic and clinical questionnaires, including age, gender, and classifications of the child’s gross motor, cognitive and communication function and manual ability. The Gross Motor Function Classification System Expanded and Revised (GMFCS-E&R), the Manual Ability Classification System (MACS) and the Communication Function Classification System (CFCS) are each comprised of a five-level classification system for children with CP, where level 5 implies the most severe function limitations [34–36]. The cognitive function was classified according the International Classification of Diseases (ICD-10) into the groups of: no mental retardation, mild mental retardation and moderate to profound mental retardation [37].

The Canadian Occupational Performance Measure (COPM) [38], adapted and shown to be reliable and valid for children [39], was used to identify important physical activities for each child and to capture the child’s self-perception over time of their performance in everyday living. Performance problems, concerns and issues around their physical activities were identified and the performance and satisfaction levels in self-care, productivity and leisure were ranked and rated from the child’s perspective on a Visual Analogue Scale 1-10. A change score of ≥ 2 was considered clinically meaningful [38]. Performance and satisfaction scores were reassessed at 8 months with a follow up at 11 months after baseline to detect changes.

Goal Attainment Scaling (GAS) [40–42] is a validated method for scoring the achievement of a goal set. Each child had their own goal set which was developed collaboratively with the child, their parents and the physiotherapist [43]. The child and their parents scored the extent to which their one to three individual goals were achieved on a five point scale ranging from -2 to +2. Baseline was set at -2, the expected level of attainment after the intervention at zero and the most favourable outcome at +2. Each step on the five point scale was considered to represent a valuable change [42].

A study specific feasibility questionnaire was designed to evaluate each part of the intervention (goal setting, questionnaires and each outcome measure) on a Visual Analogue Scale 1-10, where one represents ‘very difficult/bad’ and 10 ‘extremely easy/good’. In addition, questionnaires for the parents and physiotherapists were designed to include fees, equipment, and transport costs of the self-selected physical activity, and time costs, such as travel time and time spent at the activity, and for assessments and other contacts during the intervention.

#### Measuring gross motor function and physical activity

The Gross Motor Function Measure 66 (GMFM-66), a 66-item clinical measure with good reliability and validity to assess gross motor function and changes over time in children with CP, was used [44, 45]. The assessments were video-recorded and independently scored by two physiotherapists.

The frequency of the child’s physical activities at school, leisure time and physiotherapy were recorded according to the questionnaire used in the National Quality Registry and CP Follow-Up Programme [46]. The International Physical Activity Questionnaire (IPAQ) is commonly used, although not validated, for self-report physical activity measures for children with CP. The IPAQ was used to estimate the time the child spent being physically active each day with light, moderate or vigorous intensity and how much time the child spent sedentary during the last seven days [47].

Accelerometers were used to measure physical activity as they have been shown to be feasible and useful for ambulatory and non-ambulatory adolescents with CP [48]. A triaxial accelerometer, ActiTrainer [49], was chosen for its ability to assess activity throughout acceleration in a lying, sitting or standing position; its weight is 51 g with dimensions of 8.6x3.3x1.5 cm. The children wore the ActiTrainer [49] on their hips during all waking hours for a seven day period according to best practices [48, 50, 51]. In addition, the participants wore a Polar^R^ heart rate monitor around their chest during all waking hours for the seven day period. Children with ≥ five hours of monitoring time on ≥ two days were included for analyses [51].

Time-use diaries were used as a complement to the accelerometers to collect information about all the activities the child participated in during a week day and a weekend day, at what level of physical intensity they classified the activities as, and where and with whom the child was when doing the activities [52].

For practical reasons, the IPAQ was used at the home visits assessing physical activity during the last seven days, while the accelerometers and time-use diaries were used during the week after the home visits.

### Procedure

In September 2013 an administrative assistant at the Child and Youth Habilitation Services sent an invitation letter with information about the study to all parents of children fulfilling the inclusion criteria; diagnosed with CP and aged 7-11 years in the Skåne region in southern Sweden (*N*= 347). The information was given to both the child and their parents, and included an easy-to-read child-appropriate version. The children and their families were invited to an inspiration day, where they could try different physical activities in the locations of the Association for Disability Sports [53] in November 2013. Approximately 80 people in all, comprising of 32 children, their siblings and parents, tried activities such as yoga, gymnastics to music, wheelchair basketball, table tennis, martial arts and a variety of activity stimulating toys. Furthermore, the assistive device Innowalk was presented [54], which offers children with severe motor impairments the opportunity to experience walking movements in an upright position. Parents of 27 children showed interest in participating in the study and all of them were contacted by the first author who then made a strategically chosen selection of eleven children to represent different demographic and clinical groups.

The physiotherapist made both a home visit and met the child with their parents at the child’s local habilitation centre for the baseline assessments. The COPM and MI-coaching led to self-selected physical activities and the written agreement including GAS between each child, their parents and the physiotherapist. Eight children selected their own physical activities, while the parents of three children with profound intellectual disability selected the physical activities. By taking the child’s and parents’ perspectives and values into account and by active listening in the form of open questions and reflections, the physiotherapist guided them towards change. The child, their parents and group leaders received participation-based MI-coaching and individual support from the physiotherapist according to each agreement. The MI-coaching varied between individuals and was used during meetings, telephone calls, e-mailings or text messages to the child and their parents.

### Statistics and calculations

IBM SPSS version 21.00 (Statistical Package for the Social Sciences v21) was used for the statistical analyses. Comparisons between base-line and the follow-up at 8 months were descriptive, and when appropriate presented as median ± interquartile range [IQR]. The Gross Motor Ability Estimator (GMAE), a free computer program from the constructors of GMFM, was used to calculate the total score, standard error, 95% confidence intervals (CI) and percentiles of the GMFM-66 [55]. The age-specific count ranges, cut-points for activity levels, developed by Freedson et al. (2005) for typically developed children at ≤100 counts/minute for sedentary time (ST), 101-499 counts/minute for light intensity (LPA), ≥ 500 counts/minute for moderate intensity (MPA) and ≥ 4000 counts/minute for vigorous intensity (VPA), were used [56]. The time each child spent in ST, LPA, MPA and VPA, measured with the ActiTrainer, is presented in Table 4 in mins/day for each intensity.

## Results

Each child chose and participated in 1-3 physical activities as presented in Table 2. For three children with profound intellectual disability, the parents chose the activity/activities. Six children who tried new physical activities in a group made new friends according to the GAS-evaluation; all 11 improved their abilities in their self-selected activities according to the COPM, with a change score of ≥2 for 34 of 38 items (Table 3). Four families chose family activities such as going swimming on the weekends or improving everyday activities such as cycling to school. There were also activities such as dancing or playing basketball families opted out of because of travel distances, inconvenient timeframes, or costs.

**Table 2:** The self-selected physical activities based on the agreement between child, parents and physiotherapist

Child	GMFCS-E&R level^a^	Individual goals	Weeks in the activity^b^	Frequency of participation^b^	Frequency of MI-coaching and support^b^
1	I	Participates in Ju-Jitsu 1x/week and has fun.	19	26	7
2	IV	1. Cycles in the neighbourhood 15 min 2x/week with support of one parent.	17	3	2
		2. Swims 1x/week together with a family member.	17	16	1
		3. Exercises according to home training programme 20 min 3x/week.	17	43	3
3	III	Participates in electric wheelchair hockey 1x/week, has fun, meets another team and wins.	22	18	5
4	IV	1. Participates in gymnastics to music with other children 1x/week.	20	12	3
		2. Exercises on the Innowalk^c^ 45 min 7x/week and enjoys it. Is more symmetrical when sitting and standing.	19	97	5
5	I	Skates one lap around the ice without falling.	10	10	7
6	IV	1. Gets actively up from sitting with foot orthoses to standing and walks a few steps 10x/day with support; stands actively up when moving from bed to chair with support.	19	1 020	3
		2. Exercises on the Innowalk 60 min 3-5x/week.	19	42	5
7	V	1. Exercises by choosing from a great selection of preparatory and fun warm-up exercises prior to each physical activity.	0	0	1
		2. Exercises on the Innowalk 45 min 7x/week.	19	42	5
8	IV	1. Participates in electric wheelchair hockey 1x/week, has fun and has friends in the group.	22	17	5
		2. Exercises on the Innowalk 45 min 5x/week.	19	84	5
9	I	Plays table tennis with the other children at the training centre and follows the rules.	19	13	1
10	II	Plays football and participates in physical activities with friends during school breaks.	18	40	2
11	II	1. Cycles to or from school 2x/week and cycles 30 min 1x/weekend.	19	16	1
		2. Swims with the family 2x/month.	19	2	1

^a^Gross Motor Function Classification System Expanded and Revised

^b^The frequency was documented through the logbook

^c^Innowalk, a motorised medical device, giving the child the opportunity to experience repetitive walking movement in an upright position

**Table 3:** Changes from baseline to the follow-up at 8 months in scores of Canadian Occupational Performance Measure, Goal Attainment Scaling and Gross Motor Function Measure 66

Child	COPM^a^		GAS^b^	GMFM-66^c^ score
	Performance	Satisfaction	Goal 1/2/3	
1	±0	+1	+1	±0
2	±0	+9	-2/-1/0	+1.6
3	+9	+3	+2	-
4	+9/+5	+9/+7	+2/+2	+7.6
5	+9	+3	+2	±0
6	+4/+6	±0/+6	+2/+1	+2.1
7	+2/+2/+4/+4/+9	+5/+3/+3/+3/+9	-2/+2	+2.7
8	+9/+9	+8/+9	0/+1	±0
9	+7	+5	+1	±0
10	+4	+9	0	+1.7
11	+2/±0	+8/±0	+1/-2	+2.0

^a^Canadian Occupational Performance Measure: 1-5 problems were identified for each child and scored 1-10; ^b^Goal Attainment Scaling, a 5-point scale ranging from -2 to +2, baseline is -2; ^c^Gross Motor Function Measure 66; - missing values

In addition, 15 out of 18 GAS scores increased clinically from baseline. However, some goals had to be adjusted during the intervention such as when the goal was achieved after a short time or the child changed their selected activity. At 8 months, 10 children performed the GMFM-66; one child had recently undergone an unplanned surgery followed by movement restrictions and therefore did not complete the GMFM-66 at the 8-month time point. In 6 children the outcome score of the GMFM-66 improved, whereas in 4 children their score remained unchanged. Changes in the scores of COPM, GAS and GMFM-66 are presented in Table 3.

The families spent 17-100 hours on the intervention, 10-19 hours for the assessments with MI-support and 1-34 hours for travel, depending on their self-selected activity. In addition, costs for the intervention varied between 120 and 6000 SEK (1 USD=8.88 SEK in 2016) among the families depending on potential fees for the activity, purchased equipment and travel costs. Time spent for the physiotherapists supporting each family and travel time related to the intervention varied but not to the same extent as time spent for the families; the physiotherapist spent between 10 and 19 hours in total for assessing and supporting each family, and between 6 and 18 hours for travel related to the intervention.

### Feasibility

The acceptability of PAP and the assessments was generally assessed as good and all families scored the overall experience of PAP with ≥ 8. Writing the agreement, COPM, GAS and performing the GMFM-66 were scored at ≥ 5, as well as writing in the logbook and time-use diaries. Parents considered COPM being a helpful assessment in identifying important activities and in detecting changes in performance and satisfaction for each child. The children enjoyed scoring by themselves or with the help of their parents. Parents and children scored the completing of the questionnaires as five on average although some scored them ≥ 8. However, completing the IPAQ by parents with support of the physiotherapist was experienced as difficult with scores 2-7. The parents found it hard to score their child as physically active in a low, moderate or vigorous level or how much sedentary time the child had during individual days or throughout a 7-day period. The families had most concerns wearing the physical activity monitors. Wearing the accelerometer on the hips disturbed two children physically while the other children accepted it well (scores 1-7). The heart rate monitor scored very low (scores 1-3) because it frequently slipped down. Two children felt that their peers were looking at them because of the accelerometer, and one child refused to use it.

### Impact on physical activity and sedentary behaviour

According to the logbooks, COPM and GAS, all of the children’s participation in physical activities increased in frequency and duration from baseline.

The results at baseline and at 8 months from the IPAQ, the time-use diaries and the accelerometers are presented in Table 4. The estimated time for each child in the different physical activity levels (sedentary time, light, moderate and vigorous intensity) varied randomly between baseline and 8 months, as well as between the estimations in the IPAQ and the time-use diaries. The measurements of physical activity and sedentary time by accelerometers showed that seven children were physically active at moderate-vigorous levels for more than 60 minutes/day at both assessments, and the median for the whole group was 84 minutes/day at baseline and 106 minutes/day after 8 months. All children spent most of their daytime sedentary and up to three hours a day in light physical activity. Their daily time spent in moderate physical activity varied between the children from less than half an hour to more than 4 hours; vigorous physical activity was rarely seen in this group. At baseline the 10 children wearing the ActiTrainer had ≥ 5 hours of monitoring time on ≥ 4 days; at 8 months the measurements for 8 children had ≥ 5hours of monitoring time on ≥ 4 days, and two children had ≥ 5 hours of monitoring time on ≥ 2 days. These results mean that the 10 measurements at baseline used 4 days of monitoring for analysis. At 8 months the 8 measurements used 4 days of monitoring for analysis and two measurements used 2 days.

**Table 4:** Minutes in participating in different physical activity levels, estimated by the International Physical Activity Questionnaires (IPAQ), documented in time geographic diaries and measured with accelerometer, presented in different levels of the Gross Motor Function Classification System Expanded and Revised (GMFCS-E&R)

			Baseline			Follow-up at 8 months		
			Level I-II	Level III-V	Level I-V	Level I,II	Level III-V	Level I-V
			(*n* = 4/5)	(*n* = 6)	(*n* = 10/11)	(*n* = 4/5)	(*n* = 6)	(*n* = 10/11)
ST^a^	min/day	IPAQ	360 [180-480]	240 [120-660]	325 [165-510]	360 [240-600]	240 [120-660]	340 [165-600]
		Diary	543 [360-630]	305 [280-430]	390 [282-554]	490 [325-563]	418 [192-519]	428 [293-553]
		Accelerometer	464 [374-563]	673 [403-699]	496 [386-689]	499 [430-596]	623 [524-687]	574 [495-618]
LPA^b^	min/day	IPAQ	300 [180-540]	540 [180-615]	360 [180-600]	360 [280-420]	540 [180-615]	420 [255-555]
		Accelerometer	119 [105-149]	84 [49-117]	109 [66-126]	128 [87-153]	95 [73-100]	128 [66-128]
MPA^c^	min/day	IPAQ	120 [60-240]	90 [60-180]	105 [60-180]	90 [60-120]	64 [25-105]	75 [67-120]
		Accelerometer	125 [69-162]	29 [20-208]	84 [28-165]	106 [87-151]	64 [25-105]	106 [67-139]
LPA^b^- MPA^c^	min/day	Diary	273 [203-434]	405 [310-520]	360 [273-439]	306 [276-461]	413 [244-613]	398 [285-543]
VPA^d^	min/day	IPAQ	30 [15-60]	15 [0-60]	25 [0-60]	45 [0-120]	0 [0-90]	38 [0-98]
		Diary	5 [0-103]	40 [8-155]	20 [0-117]	45 [14-76]	25 [0-113]	45 [0-75]
		Accelerometer	2 [0-4]	0 [0-5]	2 [0-5]	1 [0-1]	0 [0-1]	1 [0-1]

Data presented as median with 25 and 75 quartile

^a^ST: sedentary time; ^b^LPA: light physical activity; ^c^MPA: moderate physical activity; ^d^VPA: vigorous physical activity

11 children filled in the IPAQ, whereas 10 children filled in time geographic diaries and 10 children used accelerometers

In the time geographic diaries LPA and MPA were merged to LPA-MPA

The cut-points used for the accelerometer were developed by Freedson et al. (2005): ≤100 counts/minute for ST, 101-499 counts/minute for LPA, ≥ 500 counts/minute MPA and ≥ 4000 counts/minute for VPA [56]

### Some scenarios

In order to exemplify the intervention, three scenarios are briefly presented by using pseudonyms in the descriptions. The GMFCS-E&R levels were IV (Robin and Andrea) and II (Kim).

Robin, aged 10 years, chose playing electric wheelchair hockey for his physical activity and hoped to learn the rules, have fun and make new friends. The physiotherapist attended two exercise sessions for observation and MI-coaching. It was important for Robin to be seen by the group leader, who was qualified in leading disabled sports groups, to get enough time to exercise both alone and together with other children with disabilities, and to get the opportunity to talk with the other children. All children in the group had varying individual capabilities, which was challenging for the group leader. Robin fell over onto one side while sitting in the electric wheelchair that he borrowed from the Association for Disability Sports [53]. Improvised adjustments made by the parents and the group leader enabled a more symmetric sitting position which helped a little for some minutes, but there were no resources for professional adjustments. Robin attended all exercise sessions which he documented in the logbook by pasting stickers. He chose a happy face to represent each session and he wrote positive comments. The GAS evaluation showed that he learned the rules of electric wheelchair hockey, had fun and made friends when playing, although he had no contact with the other children in the group in between the exercise sessions.

Kim, aged 9 years, wanted to dance or join a sports group together with other children with disabilities. The physiotherapist explored the possibilities and suggested several activities, but none fit into the family life. The times were inconvenient either for Kim, because it would be too late on a weekday, or the parents who could not make it from work on time for the activity. By identifying areas of interest through COPM and MI change talk, the written agreement between Kim, her parents and the physiotherapist then became about choosing an everyday activity. She wanted to cycle to school with one of her parents twice a week and once every weekend. Kim filled in the logbook at the weekends, whereas the parents did it during the week. At 8 months they felt that cycling to school twice a week as a habitual physical activity had become a routine that they wanted to continue.

Andrea, aged 11 years, had shown a deteriorated walking ability during the last months before the intervention started and needed help from two people for all transfers, i.e. from sitting in the wheelchair to the toilet. In addition, Andrea was very restless, showed self-destructive behaviour and only slept for short periods at night. In order to increase physical activity and decrease sedentary time for her at home, Andrea’s parents chose the motorised assistive device Innowalk [54] for her physical activity. She used the Innowalk to experience a walking movement in an upright weight-bearing position every weekday and often twice a day at the weekends. The acceptability was good: Andrea and her parents liked the Innowalk and it became a daily routine. At 8 months the scores of the GMFM-66 had improved significantly and she walked 8 steps independently with just with one person behind her. The evaluation of the goals, according to GAS, showed that she walked with one person indoors from one room to another, the self-destructive behaviour had decreased and her sleeping pattern had become more regular. An unexpected perceived outcome was that the intervention had a positive effect on her constipation. The Innowalk was returned at 8 months; at 11 months the outcomes had not maintained, according to the parents’ statements.

## Discussion

This intervention aiming to evaluate the feasibility and effectiveness of PAP for children with CP showed no drop-outs. Overall, PAP was considered feasible and widely accepted among the children and their parents, even though some children did not like to wear the heart rate monitor and one child refused to wear both the accelerometer and the heart rate monitor. The IPAQ was difficult to use and is so far not validated for children with CP. Through the intervention the children’s participation in physical activities increased, and several children made new friends during their self-selected physical activities. The outcome scores according to satisfaction and performance (COPM) and to individual aims (GAS) increased overall, and clinically meaningful improvement of GMFM scores was found for six children during the intervention.

However, the intensity levels of physical activity measured with the accelerometer did not show an increase for all children. Eight children met the global recommendations of 60 minutes of moderate to vigorous PA daily, some of them by a wide margin. Two children were very restless at baseline and constantly moving around. Despite increasing volitional functional physical activity, their total physical activity decreased at 8 months. The restlessness contributed significantly to their total physical activity levels at baseline as measured with the accelerometer. These results highlight the importance of taking the individual quality of the child’s physical activity into account among children with CP.

Having a written agreement between each child, their parents and a physiotherapist and combining this with individual support during the child’s participation in the self-selected physical activity was given high scores by children and parents according to the evaluation questionnaires. There is evidence that PAP can work for adults in need of a more active lifestyle [28]. Varying exercise programmes, home-based physiotherapy and counselling have not shown long-term effects for children with CP [29–31], but to our knowledge, no research on PAP for children with CP has been done. The time and costs each family spent for PAP differed widely, between 17 to 100 hours and 120 and 6000 SEK. Type of selected physical activity, such as expensive group activities far away from home or performing active transfers from sitting to standing several times a day, may explain some of these differences in time and associated costs.

The combination of PAP and MI is aimed at encouraging a lifestyle change provided that the participant also has a desire for change [28]. Therefore, MI was used throughout the study period and enabled the physiotherapist to coach each child and their parents individually. MI change talk is based on each individual’s readiness for change. This strategy is consistent with suggestions from another study [57] where three stages of changing attitudes towards physical activity for children with CP and their parents were identified. According to the authors it is important to be aware of whether the child and their parents are in a pre-intention stage, implying that they are not fully informed and have not yet decided to act, or whether they are in the intention stage, having decided to engage in physical activity behaviour but have not yet started to act. Being in the action stage implies acting out their intentions regarding physical activity. It seems reasonable to assume that the parents in the present study were motivated and might have been in the intention or the action stage when they signed up to participate in the study. However, their child might have been in a different stage, which became obvious when writing the agreement based on MI, COPM and GAS. Children and parents being in different stages of changing attitudes might imply a challenge for the physiotherapist to use the MI change talk, which was not measured in this study but should be considered in future research.

The COPM proved to be a useful tool for the MI change talk by identifying activities that were important for the child, which increased the child’s motivation for participating in the physical activities. The results of another study confirmed the importance of engagement and motivation when young people with CP were physically active [58]. The authors concluded that participation in activities was a key factor for motivation, which is consistent with the findings of interviews with children with CP [20]. The children in the present study often set their goals based on a social participation-level, and were looking forward to making new friends during their self-selected physical activity. Palisano et al. [59] found that real-life experiences enable children to optimise their participation and self-determination. The families increased their awareness about accessibility of physical activities and locations and the children got the opportunity to try self-selected activities with appropriate support. On the other hand, not all of the physical activities that the children most wanted to do fit into their family lifestyle or were accessible in their local community. The COPM with MI led to other activities that fit the family better, and we wrote an agreement with goals according to GAS. Appropriate timeframes, weekday and accessibility of the physical activities, competent leaders, the opportunity to become friends with other children, and the costs of the activity were all important requirements when the children selected their physical activities. Children with more severe motor limitations were dependent on adjustments of assistive devices and on the availability of assistive devices, such as the Innowalk, in order to participate in physical activities. Due to a lack of optimal conditions some children could not perform their self-selected activity in the best way, such as the child that played electric wheelchair hockey in an asymmetric sitting position.

### Strengths and limitations of the intervention

The intervention was carried out and evaluated in real-world, non-ideal conditions, which according to Bowen et al. [27] implies the possibility to evaluate its effectiveness. This non-clinical setting, combined with the choice to participate in organised group activities or in everyday activities, can be seen as facilitating factors for PAP. The COPM, GAS and GMFM-66 are sensitive to change, which our evaluations confirmed. The GMFM-66 has been criticised for not measuring changes for children with mild CP- they already score 100 % at baseline [45]. The COPM is an individualised and evidence-based outcome measure [38, 39]. The COPM and GAS might be sufficient to evaluate change over time; on the other hand the GMFM-66 may add a dimension of measuring the child’s gross motor function that can be useful when selecting physical activities, setting goals and supporting the child in their participation. Two of the authors were active in the intervention which might imply a bias. In order to reduce bias, these issues were discussed during the whole study process in the research group.

Documenting the participation for their self-selected physical activities in their individual logbooks was considered enjoyable and feasible by the children. However, completing the IPAQ was difficult, using the heart rate and physical activity monitors was experienced as inconvenient, and completing time-use diaries when wearing the physical activity monitors was time consuming. In addition, the IPAQ was used at the home visits while the time-use diaries and physical activity monitors were used during the week after the home visit. That was done due to practical reasons as it would have implied additional home visits to get all measurements in the same week. Synchronising the use of these instruments is recommended when using them. A feedback from baseline assessments of physical activity intensity should be given before the second assessments. These second assessments should be done during the period when the child participates in the physical activity, which might improve the motivation for using the heart rate and physical activity monitors in combination with time-use diaries and the IPAQ. For discussions concerning change and for counselling throughout the intervention MI was used. Other studies have found MI useful for increasing physical activity in adults with chronic health conditions when integrated with other treatments [33]. On the other hand, a systematic review [60] could not show effects of MI on physical activity as an independent intervention in adults. Therefore, it seems reasonable to integrate MI into PAP, which is supported by our results.

To improve the practicality and likelihood of implementation, modifications to the study which we recommend include not performing all assessments with the family at the same meeting.

## Conclusions

The intervention PAP seems to be feasible and effective for children with CP, involving both every day and organised physical activities to promote an active lifestyle through increased participation, motivation and engagement in physical activities. In future studies, reducing sedentary behaviour and increasing light and moderate physical activity for children with CP could be a possible focus. Further research of PAP for children with CP is needed, preferably in a randomised controlled long term trial and including health economic analysis to show costs and benefits.
